# Obtaining extremely large and accurate protein multiple sequence alignments from curated hierarchical alignments

**DOI:** 10.1093/database/baaa042

**Published:** 2020-06-08

**Authors:** Andrew F Neuwald, Christopher J Lanczycki, Theresa K Hodges, Aron Marchler-Bauer

**Affiliations:** 1 Institute for Genome Sciences; 2 Department of Biochemistry & Molecular Biology, University of Maryland School of Medicine, 670 W. Baltimore Street, Baltimore, MD 21201, USA; 3 National Center for Biotechnology Information, National Library of Medicine, National Institutes of Health, Building 38 A, 8600 Rockville Pike, Bethesda, MD 20894, USA

## Abstract

For optimal performance, machine learning methods for protein sequence/structural analysis typically require as input a large multiple sequence alignment (MSA), which is often created using query-based iterative programs, such as PSI-BLAST or JackHMMER. However, because these programs align database sequences using a query sequence as a template, they may fail to detect or may tend to misalign sequences distantly related to the query. More generally, automated MSA programs often fail to align sequences correctly due to the unpredictable nature of protein evolution. Addressing this problem typically requires manual curation in the light of structural data. However, curated MSAs tend to contain too few sequences to serve as input for statistically based methods. We address these shortcomings by making publicly available a set of 252 curated hierarchical MSAs (hiMSAs), containing a total of 26 212 066 sequences, along with programs for generating from these extremely large MSAs. Each hiMSA consists of a set of hierarchically arranged MSAs representing individual subgroups within a superfamily along with template MSAs specifying how to align each subgroup MSA against MSAs higher up the hierarchy. Central to this approach is the MAPGAPS search program, which uses a hiMSA as a query to align (potentially vast numbers of) matching database sequences with accuracy comparable to that of the curated hiMSA. We illustrate this process for the exonuclease–endonuclease–phosphatase superfamily and for pleckstrin homology domains. A set of extremely large MSAs generated from the hiMSAs in this way is available as input for deep learning, big data analyses. MAPGAPS, auxiliary programs CDD2MGS, AddPhylum, PurgeMSA and ConvertMSA and links to National Center for Biotechnology Information data files are available at https://www.igs.umaryland.edu/labs/neuwald/software/mapgaps/.

## Introduction

Certain protein sequence analysis methods require as input a large number of multiply aligned sequences. This includes, for instance, direct coupling analysis (DCA) (1–7), which uses statistical modeling to predict 3D interacting residue pairs. DCA typically requires at least a thousand and, ideally, tens of thousands of aligned sequences. Larger multiple sequence alignments (MSAs) are required for deep neural networks and for other machine learning algorithms, such as Bayesian partitioning with pattern selection (BPPS) (8). BPPS, like DCA, identifies statistical correlations in an MSA but, unlike DCA, focuses on sets of subgroup-specific co-conserved residues associated with functional specialization rather than on pairwise correlations. For BPPS, at least tens of thousands of aligned sequences are required to obtain accurate subgroup models, each of which might be based on at least a hundred sequences. This process defines sub-MSAs that are useful for performing DCA on superfamily subgroups (9).

Of course, to best distinguish signal from noise, sequences also need to be aligned accurately. In this regard, automated MSA programs are far from optimal (10–14) due to the unpredictable nature of protein evolution. Consider, for example, a conserved arginine residue that is just beyond a guanine-binding NK.D motif in Ran, Rab, Ras and Rho GTPases and that forms a salt bridge with a conserved acidic residue (15). Within some of these GTPases, a deletion (of up to three residues) directly precedes this arginine and an insertion (of up to 15 residues) directly follows it (16)—making it more or less impossible to align correctly without manual curation. Moreover, distinct subgroups within a large superfamily typically harbor insertions and deletions relative to other subgroups thereby confounding MSA methods. For these reasons, the National Center for Biotechnology Information manually curates hierarchical MSAs (hiMSAs) for hundreds of widely distributed and diverse protein domain superfamilies. RPS-BLAST (17) searches against this conserved domain database (CDD) (18) identify domains within a query sequence and the subgroup to which each domain belongs.

Curated hiMSAs have other useful applications as well, including serving as benchmark sets (14). Another application, which we highlight here, is as queries for the MAPGAPS (Multiply-Aligned Profiles for Global Alignment of Protein Sequences) program (16) to thereby create extremely large and accurate MSAs. Here we announce the public availability of (i) a set of CDD hiMSAs, (ii) a suite of programs for creating from each hiMSA an extremely large MSA and (iii) a set of MSAs obtained in this way. The software package contains MAPGAPS (v2.1) and utilities for format conversion, taxonomic annotation and the removal of undesirable sequences from the output MSA. Phylum annotation and species identifiers are particularly useful for both DCA and BPPS. For DCA, species identifiers facilitate prediction of residue 3D contacts across a protein–protein interface by identifying, for each species, pairs of interacting proteins. For BPPS, this facilitates both co-analysis of interacting proteins and characterization of subgroup phylogenetic diversity to confirm that residue patterns are due to persistent evolutionary constraints rather than merely due to recent common descent. Of course, having an extremely large and accurate MSA also provides additional statistical power for DCA and BPPS and is necessary for machine learning based on deep neural networks.

### Availability of CD hiMSAs and of corresponding MSAs

Version 3.17 of the CDD provides over 57 000 position-specific scoring matrices and MSAs corresponding to ancient conserved domains (CDs) for individual protein subgroups that span diverse organisms. Subgroup alignments are kept consistent throughout each superfamily hierarchy, which allows each of these to be mapped onto its typically less extensive `parent’ alignment. CDD subgroups correspond to major branches within sequence trees. CDD alignments are manually curated based on both sequence homology and structural superposition. Superfamily members share a common structural core but often perform diverse biochemical or cellular functions. Each sequence assigned to a given subgroup is also assigned (either explicitly or implicitly) to its parent subgroups. For each subgroup, a consensus sequence is defined based on the most frequent residue per column after weighting for sequence redundancy.

Here we announce the availability (at ftp://ftp.ncbi.nih.gov/pub/mmdb/cdd/hiMSA) of 252 curated CDD hiMSAs and of MSAs derived from these. These hiMSAs consist of at least 10 nodes, and their derived MSAs contain at least 1000 sequences after removing sequence fragments matching <75% of the aligned columns and reducing sequence identities to ≥98%; the average length of these MSAs is 157 columns and contain an average of 50 nodes and 106 109 sequences. Each superfamily hiMSA comes as a set of text files in mFASTA format along with template files that define how to globally align each subgroup MSA against the other subgroup MSAs from the same superfamily. Each superfamily’s file is in a subdirectory named after its identifier (e.g. cd08372). The numbers of sequences in the corresponding MSAs are given in Table S1. Although existing methods may align a small number of sequences with comparable accuracy, aligning the numbers of sequences obtained here overwhelms these programs. This includes two recent programs designed to align larger numbers of sequences (19, 20); these failed to align most of the sequence sets in Table S1 using the maximum amount of RAM available on our systems.

### Using hiMSAs to obtain MSAs

**Figure 1:**
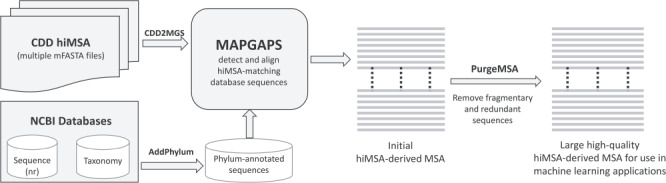
Steps required to create a large, high-quality MSA using the CDD hiMSAs and programs described here.

The software needed to construct an extremely large, accurate and taxonomically annotated MSA from the corresponding hiMSA is available at http://www.igs.umaryland.edu/labs/neuwald/software/mapgaps/. The procedure, which is outlined in Figure 1, involves the following. (i) The CDD2MGS program converts an mFASTA formatted CDD hiMSA into MAPGAPS’s input format. (ii) Using the hiMSA as a query, MAPGAPS searches a large sequence database, such as the FASTA-formatted BLAST nr database (found at ftp://ftp.ncbi.nih.gov/blast/db/FASTA/). (The AddPhylum program can be used to label nr sequences by phylum and kingdom, which is useful for BPPS.) It is recommended to first split a large sequence database into smaller FASTA files using the program fasplit. MAPGAPS can then be run in parallel on each of the split files. Splitting the current version of nr into subsets of 250 000 sequences results in about 750 sub-files. After all subsets have been processed, the MAPGAPS output files are concatenated into a single file. (iii) The program PurgeMSA merges the output files while removing short sequence fragments and redundant sequences. We suggest removing those sequences matching <75% of the aligned columns and retaining all but one sequence among those sharing ≥98% sequence identity. In addition to the default MAPGAPS cma-formatted MSA, which requires relatively little memory and disk space, the ConvertMSA program can convert this into mFASTA-format, which, however, requires significantly more storage space and for large superfamilies may exceed available memory. ConvertMSA can also convert mFASTA-formatted MSAs into cma format and cma-formatted MSAs into rich text format, such as in Figure 3.

The MAPGAPS algorithm has been described in detail previously (16) but is summarized here as follows: MAPGAPS takes as input files (named using an arbitrary prefix): (i) an array of MSAs (in cma format with file extension *.cma) corresponding to the root, internal nodes and leaves of the CD hierarchy; (ii) a `template’ MSA (in cma format with extension *.tpl) that aligns the consensus sequences to each other and to the root; and (iii) an optional heuristic pruning tree (in depth-first traversal integer array format (16) with extension *.dft). The template consists of a set of multiply aligned consensus sequences—one sequence for each profile with the first sequence representing the consensus sequence for the template itself. Prior to a search, MAPGAPS generates profiles (i.e. position-specific scoring matrices), one for each MSA within the hiMSA. Using these profiles as the `query’, MAPGAPS uses PSI-BLAST (21) heuristics to search for database sequences with a significant match against at least one of the profiles. It also prunes the search space as follows. First, it scores each database sequence against a profile of the entire superfamily. Those sequences that attain a specified `threshold trigger score’ are then compared against the other profiles. Because the profiles are arranged as a tree, only those sequences that obtain at least a threshold trigger score against a parent profile are searched against the associated child profile(s). Each sequence that has a significant score against at least one profile is assigned to its highest scoring profile. Of course, multiple copies of a domain within a single sequence may each be detected in this way. Alternatively, a single copy of a domain may be detected as two or more short regions of sequence similarity; MAPGAPS combines these into a single, longer region of similarity. Finally, MAPGAPS uses the template alignment to globally align all of the matching database sequences to each other. In this way, as long as the curated alignments within a hiMSA accurately represent the subgroups within the superfamily, homologous residues, which might otherwise be impossible to align correctly, can be aligned with accuracy comparable to that of the curated hiMSA (16).

### Illustration with EEP and PH domains

**Figure 2:**
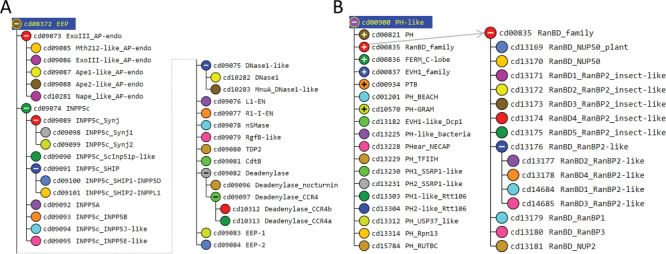
CDD hierarchies used here to create very large MSAs. **A**. Hierarchy for EEP domains (cd08372). **B**. Hierarchy for PH domains (cd00900). The subtree is shown for the RanBD family; other (+) nodes may be expanded in a similar manner.

We illustrate this process using hiMSAs for the exonuclease–endonuclease–phosphatase (EEP) and pleckstrin homology (PH) domain superfamilies. The EEP superfamily is functionally diverse and includes the ExoIII family of apurinic/apyrimidinic endonucleases, inositol polyphosphate 5-phosphatases, neutral sphingomyelinases, deadenylases, bacterial cytolethal distending toxin B (CdtB), deoxyribonuclease 1 (DNase1) and the endonuclease domain of the non-LTR retrotransposon LINE-1. The EEP hiMSA (cd08372) consists of 35 nodes (Figure 2A). A MAPGAPS search of the nr, translated EST (22) and environmental nr databases using the EEP hiMSA as the query generated an alignment of 223 493 sequences. Removing sequence matching <75% of the columns in the root node MSA or sharing ≥98% sequence identity with other sequences left 108 153 sequences. This MSA is sufficiently large to perform both DCA and BPPS (as was described in (8)). A representative alignment of 33 sequences among those most distantly related in the MSA is shown in Figure S1. Despite weak similarity and an abundance of indels, the motif characteristic of this superfamily is generally well aligned for these sequences.

**Figure 3:**
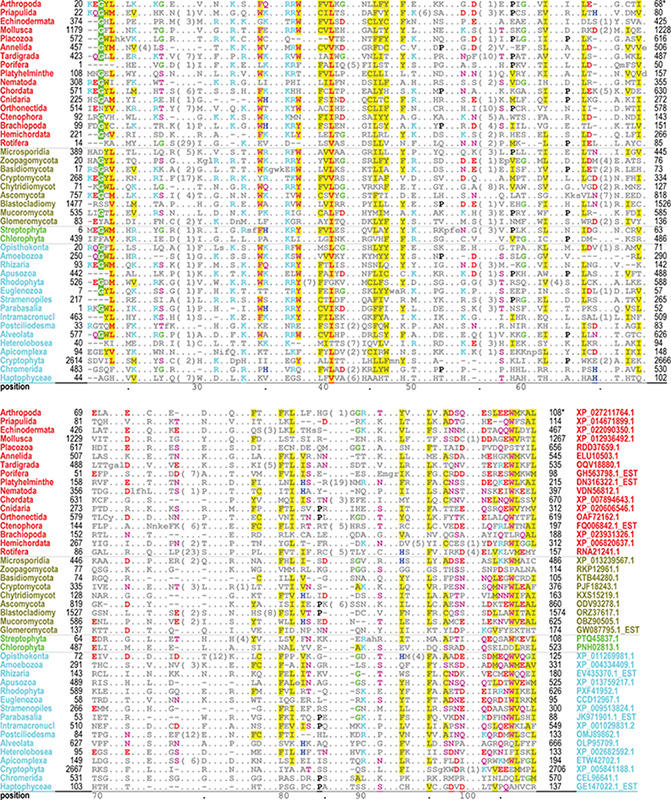
Alignment of 42 representative, distantly related PH domains from distinct phyla and sharing ≤25% identity. Despite the weak similarity and an abundance of indels, conserved residues characteristic of this superfamily are generally well aligned. Residues generally conserved in `all’ PH domains are colored as follows: acidic residues, red (without highlighting); basic residues, cyan; hydrophilic residue, pink; histidine, glycine and proline, blue, green and black, respectively; hydrophobic and aromatic residues, red (highly conserved) or gray with yellow highlighting. Identifiers for phyla (left column) and sequences (right column in lower aligned region) are color coded by taxa as follows: metazoan, red; fungal, dark yellow; plant, green; protozoan, cyan.

The PH domain hiMSA (cd00900) consists of 335 nodes (Figure 2B). We chose this domain because it is one of the most difficult to align correctly and the superfamily contains too many member sequences to align using conventional methods: even after removing fragments with <75% matches and reducing redundancy to <98%, the MAPGAPS-generated MSA consisted of 130 774 sequences. A representative MSA consisting of 42 sequences from distinct phyla is shown in Figure 3. Each of these sequences shares <25% identity to the other sequences and thus are among the most difficult to align correctly. Despite the weak sequence similarity and an abundance of indels, the motifs characteristic of this superfamily are generally well aligned.

**Figure 4:**
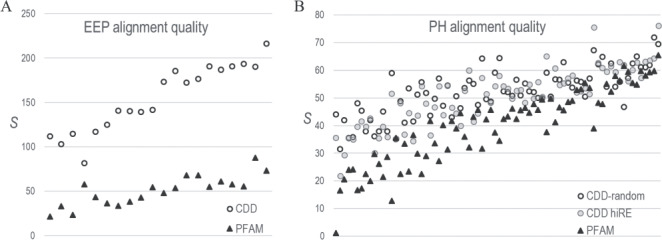
CDD versus PFAM alignment quality. *S*-scores estimate the statistical significance of the correspondence between pairwise correlations in an MSA and 3D residue contacts in available structures. (For pdb identifiers see supplementary data S1 file.) Higher-quality MSAs should yield higher *S*-scores. **A**. Comparison of CD08372-MAPGAPS versus PF03372_full EEP domain MSAs based on 20 EEP protein structures. **B**. Comparison of CDD00900-MAPGAPS versus PF00169_full PH domain MSAs based on 76 protein structures. Two CDD MSAs were analyzed: one very diverse sub-alignment of randomly sampled sequences (average column relative entropy = 0.26 nats) and another less diverse sub-alignment (denoted as hiRE) consisting of sequences very similar to those in the PFAM MSA (avg. relative entropy = 0.83 nats). The PFAM MSA was of intermediate diversity (avg. relative entropy = 0.69).

### Comparisons with large PFAM alignments

To evaluate the quality of our EEP and PH domain MSAs, we compare them with corresponding PFAM (23) MSAs: PF03372_full (Exo_endo_phos) and PF00169_full (PH), which consist of 42 099 and 50 175 sequences, respectively. Because available benchmark MSAs typically contain only a few sequences, they are inadequate for evaluating the quality of these very large MSAs. Instead, we apply the STARC program (9), which is based on the following principle: over evolutionary time substitutions at one residue position often result in compensating substitutions at other positions in order to maintain critical interactions. For proteins sharing a common structure, such contacts generally produce correlated substitution patterns between residue pairs at distinct positions in an MSA. STARC first applies direct coupling (DC) analysis (24) to measure these correlations and then estimates the statistical significance, expressed as *S* ≡ −log_10_ (*P*-value), of the correspondence between the highest DC-scoring column pairs and 3D contacts within protein structures (9). Because this correspondence depends upon properly aligning homologous residues, *S*-scores provide a direct measure of MSA quality.

To apply this approach, we first ensured that both the PFAM and the CDD-generated MSAs share the same numbers of aligned columns and of sequences and are preprocessed in the same way. For the EEP domain, ensuring the same number of columns merely required trimming the N- and C-terminal ends of the CDD MSA by one and seven residues, respectively—after which both MSAs consist of 233 aligned columns. For the PH domain, this involved trimming the N- and C-terminal ends of the PFAM MSA by two and five columns, respectively, and removing columns corresponding to insertions relative to the CDD MSA. The resulting EEP and PH MSAs consist of 233 and 89 columns, respectively. (See Figures S2–S4.)

For all MSAs, we again removed sequences with deletions in >25% of the columns and all but one sequence among each subset of sequences of unknown structure sharing ≥98% identity. Finally, we randomly removed sequences from the larger, CDD MSAs to bring the number down to that of the proteins (of unknown structure) in the PFAM MSAs, after which we added back in those proteins of known structure that are also present in the PFAM MSAs. The resulting EEP MSAs, which both consist of 26 823 sequences, share comparable sequence diversity: the average column relative entropies for CDD and PFAM were 0.54 and 0.56 nats, respectively. However, for the PH MSAs, which consist of 22 585 sequences, the CDD and PFAM average column relative entropies were 0.26 and 0.69 nats, respectively. To eliminate possible bias due to the higher CDD sequence diversity (i.e. lower relative entropy), we created a second CDD PH MSA consisting of the 22 585 sequences most similar to the PFAM sequences. This CDD MSA’s average column relative entropy was 0.83 nats. *S*-scores for the EEP and PH domain MSAs indicate that all three CDD-MAPGAPS MSAs are of higher quality than the PFAM MSAs (Figure 4), while also being substantially larger.

## Discussion

Currently, large MSAs are typically constructed using query-based iterative alignment programs, such as PSI-BLAST (21) and JackHMMER (25). Because these programs align sequences relative to a single query-defined profile, they tend to misalign sequences distantly related to the query. MAPGAPS can largely avoid this problem by using as the query a curated hiMSA that well represents each subgroup within a superfamily, as is the case for EEP and PH domains. For hiMSAs with poor subgroup representation, one can use BPPS to define new subgroups given a sufficiently large input MSA derived from the hiMSA. By facilitating curation of additional subgroups iteratively, this approach also aids further refinement and expansion of CDD hiMSAs. Although we focus here on searches of the nr and EST databases, inclusion of other protein data sources, such as translated sequences from the Transcriptome Shotgun Assembly (26) and IMG/M (27) databases, will allow construction of even larger MSAs that can lead to even better coverage of protein subgroups. For example, DCA was substantially improved recently by including a vast amount of IMG/M sequence data (28). This approach should be particularly useful for protein sequence machine learning applications, such as recent methods based on convolutional neural networks (29) or restricted Boltzmann machines (30), which require extremely large and accurate MSAs.

## Supplementary Material

neuwald_FigS1_baaa042

neuwald_FigS2_baaa042

neuwald_FigS3_baaa042

neuwald_FigS4_baaa042

neuwald_TableS1_baaa042

neuwald_TableS2_baaa042

neuwald_DataS1_baaa042
