# Supplementary material for: Obtaining extremely large and accurate protein multiple sequence alignments from curated hierarchical alignments
Source: Database (Oxford). 2020 Jun 8;2020:baaa042. doi: 10.1093/database/baaa042 (PMC7297217; doi:10.1093/database/baaa042)
Supplement: neuwald_FigS1_baaa042 [file neuwald_figs1_baaa042.docx]

## Extremely Large and Accurate Multiple Sequence Alignments based on Conserved Domain Database (CDD) Hierarchies

## Andrew F. Neuwald, Christopher J. Lanczycki, Theresa Hodges, and Aron Marchler-Bauer

### **Supplementary data**

**Figure S1**. Alignment of 33 representative, distantly related EEP domains (<30% identity) from distinct phyla and selected from among the CDD CD08372-MAPGAPS alignment. Despite the weak sequence similarity and an abundance of indels, the motifs characteristic of this superfamily are generally well aligned. Sequence identifiers are color coded by taxa as follows: metazoan, red; protozoan, cyan; fungal, dark yellow; plant, green; bacterial, purple; archaeal, blue.

**ETO10795.1**  32 **VCSWNT..LYK.........QW.....Vmkedyptiedehrtwe.........FRLP..RLR.....QKM..SDI.NA..DILCLQE...ID...K.......ST.FKEDFGDYLKK.....T** 98

**PRP87210.1**  61 **ILSWNI..LAD.........LS.....Sgsnetdptkligqvrteyllwe...NRRE..RLY.....DRV..DEL.RT..DVVCFQE...VE...E.......VI.YHGDLEERMNE.....L** 133

**GT049204.1_EST**  55 **VVSFNI..LSQ.........TC.....Leqhrhdrypnigahecgtwn.....YRLR..RIS.....HLL..LSM.MP..SIICLQE...VD...L.......DPiIIKDLEKQFAH.....H** 126

**XP_027023568.1**  267 **VVTYNI..LAE.........IY.....Sqtelsktvlypycapyaleld....YRQN..LIK.....KEL..SGY.NA..DIICLQE...VD...K.......GV.FGDSLVPALDA.....F** 338

**OAF70069.1**  571 **VVSYNI..LAQ.........GY.....Tqlsnfpwlpyshlrni.........TRLP..ILI.....DEI..RAL.NA..NVVCMQE...VE...G.......AF.LKSHLEGAMAM.....L** 637

**XP_001320460.1**  151 **-LSYNI..LAP.........YC.....Vrpdrfpfsppkylnad.........QRIA..LIE.....EQI..IEF.PV..SIVCLQE...VE...G.......SV.YKEKLEPFMHE.....R** 216

**RMZ57518.1**  510 **VLSYNM..LTD.........AP.....Graktlravspealews.........HRWR..LLS.....TQI..SGS.DA..DIICLQE...VS...M.......HS.WQELV-DLLSS.....R** 575

**XP_013759925.1**  7 **VMSYNV..LAQ.........VH.....Igkryaalgeevtswd..........VRRP..KIL.....DEI..LRL.GP..DVAACQE...MP...I.......PK.FQADLASPLAA.....A** 72

**OLP93574.1**  1557 **CMSYNV..LLPnskdgwwiyKY.....Ykdshgphtswp..............ERQA..LLA.....QQI..ARV.QP..DVLCLQE...VS...E.......LS.FDEDFR-FLSE.....Q** 1626

**XP_018711147.1**  1 **-MTYNVfaLCG.........HW.....Rfrdsngfpmih..............FRYP..ILT.....HEI..GTY.RP..DIVCLQE...ID...R.......RE.TRK-LQERLGM.....L** 62

**XP_002682001.1**  196 **VMSYNV..LAQ.........SY.....Tsthynyckpyigfg...........VRTP..KIV.....SLV..DSI.QA..DIQMYQE...ME...K.......DM.LQQLTKPHHHV.....-** 259

**OJY24566.1**  4 **VVSWNV..LAD.........AY.....Vrreyyphttpdvldra.........KRRR..AVA.....DRI..ARLgDV..EVLCLQE...AD...S.......AL.FE---LAQEKL.....P** 68

**RKP20909.1**  21 **VLTYNI..LSP.........KL.....Snigtmkkiskenllwd.........RRCP..LIT.....QEI..F--.--..DVFGLQE...VE...P.......AH.YMTILSKKFDE.....-** 82

**XP_005778738.1**  228 **------..---.........--.....-hsycdpsltagg.............LRLP..RIL.....SEV..LAA.SP..DVAALQE...VD...V.......SW.ARRFWEPTLAA.....A** 278

**XP_001749499.1**  602 **ILSYNV..LAD.........CY.....IredlarpevraasfpgkddqcllwkHRWP..RIQ.....SVV..RRS.KA..DVICLQE...VE...Y.......AM.FEHVYCPFMRR.....Q** 677

**OGN55659.1**  23 **LVTYNI..LSD.........DHimnglYdhvneeilkwq..............NRRD..KIV.....ERI..KNL.DP..DVICLQE...LN...T.......TS.FAYF---KEAL.....E** 86

**WP_009782430.1**  56 **VITYNI..GYL.........SG.....Ltnnqavkrekvlfd...........ENLE..LAI.....AAL..KP-.-FnaDFIALQE...VD...F.......AS.QRSYDVNQVQElatalK** 125

**PCJ61273.1**  8 **LVSYNL..WNI.........ER.....Wp........................DRES..ALV.....HFL..KTF.IP..DIFCVQE...LR...P.......ET.LHCI---DQTL.....I** 56

**XP_001022028.3**  50 **IMQYNV..LAP.........CY.....Typnlypdctkqdlewn.........ARLD..LLI.....KEI..KFV.DP..TILCLQE...TQ...L.......DT.LYDLNDKLREI.....F** 116

**OMJ92191.1**  5 **IGTFNI..LNT.........SC.....Rye.......................ERKI..ELK.....RTL..SEL.KC..DIIGLQE...-L...N.......FQ.INPE---LFDL.....S** 53

**XP_005836066.1**  6 **VVSYNI..LCS.........SL.....Apadrfrnckpenldaa.........VRLP..RILlkmeqEES..RVE.KE..AIICLQE...VG...-.......--.--KFWSKELES.....-** 71

**XP_023241899.1**  749 **------..---.........--.....-.........................---E..KLL.....TEL..KRT.NS..DFICLQE...I-...P.......KK.YYDILESDLNS.....L** 783

**RPD82043.1**  62 **VMTWNL..LAQ.........SL.....Vrrelfptsdclkag...........QREH..MLY.....REI..LSH.NA..AICCLQE...V-...-.......DR.TEKLF-PVLEK.....A** 123

**KXJ22915.1**  6 **--TFNI..GGA.........GS.....Xd........................NRKE..IIK.....KFV..DEH.RP..SLLFIQEhpwVK...D.......NM.FKHI---TGKF.....N** 55

**ORY43732.1**  25 **VMSYNG..LAD.........TL.....Lhrhphlyrhlphnllawa.......VRVK..AIG.....DEI..SRF.SP..DVVCLQE...VE...P.......SA.FKNDFQKMLEK.....-** 92

**WP_053597247.1**  29 **---WNS..EVE.........KE.....N.........................RKNRviKLI.....NFL..SNK.NP..EIIVLLEfd.SS...T.......KQ.IEFL--NFINE.....K** 78

**RJQ10437.1**  35 **VVSLNT..WKA.........DG.....Pyr.......................ARLA..VMA.....EQLraLE-.-P..DVVCLQE...AFvavD.......GS.LSTPKALAAAL.....G** 90

**WP_082554083.1**  281 **VVSWNL..KAP.........EL.....Vgkwpawv..................IRRA..RQV.....KLL..LAM.AA..SVLLVQE...AGg..P.......SK.VKWY---DKAL.....D** 336

**OAQ31744.1**  140 **VMTYNL..LSN.........RL.....Alqnqhlykrcsksdikwe.......NRSK..LLM.....QEL..RSQ.ML..DIYCLQE...ID...R.......EH.YVSFFQPTFRE.....W** 208

**XP_022302311.1**  241 **LVSLNV..NGL.........Q-.....-.........................RKRD..KLR.....KWI..TDG.QF..GIVFLQE...TH...F.......VA.GTNS--ENECM.....P** 287

**CUF62170.1**  43 **------..---.........--.....-ye.......................QRTV..LHL.....QHI..EAL.RP..SIVVLEE...VD...GpltapptVP.TLPQ--RLINS.....G** 88

**WP_010876562.1**  5 **VLTWNL..NRA.........S-.....-.........................YRRR..KLW.....SYM..GEL.EF..DAGFFQE...VY...-.......--.---------MI.....P** 41

**EWM30668.1**  195 **------..---.........--.....-.........................----..---.....---..---.DT..DIICLQEff.VQ...D.......EG.YTTLFSSSLAD.....A** 222

**position**  . 10 . 20 . 30 . 40 . 50

**ETO10795.1**  99 **GnYE..WN.VN....L.T.KKN................KI..MM...S....N....AIAWKKNKF..........K......LIW......HEWR........S.........RTMMmlfqh** 144

**PRP87210.1**  134 **G.YD..GV.MQ....S.K.---................VH..HN...G....N....AIFFDREKF..........K......L--......----........-.........--RF.....** 161

**GT049204.1_EST**  127 **G.YV..MI.LQd...P.K.KSSh...............LP..LF...V....T....AILFLSDEF..........E......LLW......HQSR........S.........RALLael..** 171

**XP_027023568.1**  339 **G.MN..GV.FK....V.K.-ER................QH..-E...G....L....ATFYRRSKF..........K......LLF......RHDI........M.........LSEAlktdt** 381

**OAF70069.1**  638 **G.YK..CV.--....-.-.SKMrsyinnkl........RY..IE...G....C....ATFYKNIKL..........K......RFN......AEKL........T.........DIFEns...** 683

**XP_001320460.1**  217 **G.FH..CT.YC....Q.K.GRAeklneafr........EM..VH...G....Q....ATFVRNSHL..........T......VIK......TECI........Q.........YRNMqqaql** 269

**RMZ57518.1**  576 **G.YH..PV.LH....T.R.SAA................QP..PD...S....CssvlAVFYKDAKF..........A......L--......----........E.........WTQE.....** 613

**XP_013759925.1**  73 **G.YT..AI.LQ....NdK.NRAe...............TH..ET...A....N....AIIFRSDLFelayenhrsrT......LVV......ALLP........R.........FGPAavipg** 129

**OLP93574.1**  1627 **G.YT..VLmHE....K.K.GRM................RP..--...-....-....ATCWKASWK..........Q......VVA......QH--........-.........----.....** 1656

**XP_018711147.1**  63 **G.YS..MV.FL....Y.H.NTV................NT..M-...-....-....AVCYRNNLF..........S......EVR......QEQS........Y.........YSVLtsndl** 104

**XP_002682001.1**  260 **-.--..-L.FK....P.K.EGR................K-..-E...G....L....AIIIDKRVW..........D......ITK......EGS-........-.........-NKLaln..** 293

**OJY24566.1**  69 **E.AT..GQ.LF....R.K.--R................GR..GE...G....C....AIFVRRS--..........-......--V......TTEP........S.........FREL.....** 101

**RKP20909.1**  83 **-.YF..SY.YI....K.R.TGD................KP..-D...G....C....CLFLRKTRF..........D......VLD......QVFV........K.........FVDHae...** 122

**XP_005778738.1**  279 **G.YA..MA.FA....P.KaSST................EG..IT...H....T....E--WPPHQG..........K......LKG......ELCT........R.........CEDAawpfr** 322

**XP_001749499.1**  678 **G.FD..--.--....-.-.--Icimqdapk........RD..VShpvG....V....ATFLRGERF..........S......VV-......----........-.........---S.....** 711

**OGN55659.1**  87 **D.FD..--.GF....F.A.KKGs...............SS..NE...G....V....GTFCKKKTF..........KetrhkaVLC......DGTS........R.........CGCT.....** 131

**WP_009782430.1**  126 **F.PV..QA.TA....I.N.WDNnyvpfptfpiyahfgkML..-S...G....Q....AILSRYP--..........-......IEL......NERI........VleqvtnkpfYENAfyld.** 190

**PCJ61273.1**  57 **N.HD..H-.-V....K.G.DQPg...............WK..IE...S....N....-IFWNREIF..........D......LIE......YG--........-.........LEDLkm...** 93

**XP_001022028.3**  117 **D.VS..VI.HR....L.K.GKS................KK..-D...G....C....TTIFKKE--..........-......---......----........E.........YEEIysvkl** 150

**OMJ92191.1**  54 **T.YTikFV.AL....P.N.PMLk...............SE..PE...FridgN....GVLIKND-I..........E......ILE......EHRL........V.........YKNN.....** 99

**XP_005836066.1**  72 **-.--..--.--....-.-.---................--..--...-....-....--FFQKRGY..........N......YIH......SGYG........E.........AYNDf....** 92

**XP_023241899.1**  784 **G.YK..GL.VSi...P.E.NKT................R-..--...G....L....GTFYKTSSF..........N......LKR......QAGV........S.........LQKIidqdl** 826

**RPD82043.1**  124 **N.YA..WV.YA....A.G.PRK................K-..-H...G....C....LIAYRKDAF..........E......CIR......RKVV........T.........YDDQeirkd** 166

**KXJ22915.1**  56 **K.RD..YK.SV....A.Y.RNAd...............KK..VN...N....C....EVFYLKNEF..........S......EVE......TDLMnefenefeR.........LSGVegsll** 109

**ORY43732.1**  93 **D.YK..GI.Y-....-.-.--Crrlg............DA..VD...G....C....ALFYRTDKI..........K......LMH......SENL........N.........FKAL.....** 131

**WP_053597247.1**  79 **G.YA..IV.--....-.-.KE-................-N..IR...Gsi..V....VVIHKIEL-..........K......VKF......ISQK........S.........N---.....** 110

**RJQ10437.1**  91 **M.NH..VT.YA....M.R.EKDrivegv..........LVpsRS...G....L....CTLSRWP--..........-......IVE......VVAR........P.........IPSDp....** 136

**WP_082554083.1**  337 **K.LG..LA.NA....G.A.TNGa...............GS..GK...W....R....VIFYRKNRW..........T......KVA......AG--........-.........LYDLpldtl** 379

**OAQ31744.1**  209 **G.YS..GV.FK....K.R.NGD................KP..-D...G....C....AIFFRNKTV..........K......AVK......LLGV........N.........FDENaf...** 249

**XP_022302311.1**  288 **D.--..LF.QNvhcfS.N.SS-................-A..SR...G....V....SILIKKSLCa.........E......IPT......KNLY........K.........S---.....** 325

**CUF62170.1**  89 **I.YN..GV.SA....G.K.-NN................EK..GD...E....T....WVLYRNDAAavst......S......LKN......DEKEgais....H.........FTVI.....** 135

**WP_010876562.1**  42 **N.EV..RW.NY....H.T.FR-................-G..EM...N....T....LLLHKDLM-..........L......DSMkrnyleISDN........H.........C---.....** 81

**EWM30668.1**  223 **-.YD..FY.LH....P.R.PSK................K-..-D...G....L....GVLLRKGKF..........R......LHG......ARGA........T.........LSRW.....** 259

**position**  . 60 . 70 . 80 . 90

**ETO10795.1**  145 **lsektkrecpqq..................MKT..D...NND.....LGANQ.....EnseakknkdekktkknqknA..Keslqlsek...Y.E........Y.....LLVCNCH.....LE** 210

**PRP87210.1**  162 **..............................SNS..R...SRA.....LLVGL.....E...................M..Ien.........N.R........L.....IVVSSLH.....LS** 190

**GT049204.1_EST**  172 **..............................NWK..-...---.....-----.....-...................N..Q...........S.K........R.....IYIGNMH.....LQ** 188

**XP_027023568.1**  382 **lhsvl.........................LEKlsT...NPA.....LKEKM.....V...................Q..Rsttlqvtv...L.Qsvsdpsk.I.....VCVGNTH.....LY** 430

**OAF70069.1**  684 **..............................MKK..Y...NINsndhrVVSKLikkmpD...................V..Iltsefefn...G.K........T.....FIVANTH.....IN** 728

**XP_001320460.1**  270 **lpsfael.......................KKH..D...ETA.....IISIV.....Q...................H..Ksap........N.L........F.....IAIVNIH.....LY** 306

**RMZ57518.1**  614 **..............................AQH..-...--G.....LLVCL.....Q...................H..Rddapcqepsa.S.S........LrssdyLHVVNVH.....LE** 652

**XP_013759925.1**  130 **mdhiaaaqdeaearvrtaaatkvadaganaPPP..D...KRA.....LKKLA.....K...................E..Vktarnkaa...R.AmrsamaphL.....VWLVCCH.....LQ** 202

**OLP93574.1**  1657 **..............................--K..D...R-T.....LIVGL.....Q...................N..Q...........A.L........L.....LFVVNVH.....LS** 1680

**XP_018711147.1**  105 **agndhgrhcv....................M--..-...---.....-MMCL.....S...................F..NedfleghsqplR.N........G.....LIIVNTH.....LP** 145

**XP_002682001.1**  294 **..............................HPQ..D...MIF.....VACRH.....K...................F..Tk..........K.T........L.....LVVC-TH.....LL** 320

**OJY24566.1**  102 **..............................VFS..D...RSG.....HVAL-.....G...................V..T...........F.A........D.....VSIVTTH.....LK** 127

**RKP20909.1**  123 **..............................CDR..D...NVG.....IIALC.....Y...................D..Kkl.........K.M........N.....AVFSTTH.....IL** 151

**XP_005778738.1**  323 **grrts.........................AFE..L...VRA.....RVCRL.....D...................L..RepppplaeylsR.R........E.....GTAAAVH.....--** 363

**XP_001749499.1**  712 **..............................AQH..R...SRA.....LLLHL.....Q...................D..Qlt.........G.H........S.....VAVGNCH.....LI** 740

**OGN55659.1**  132 **..............................PTQ..P...---.....--ALF.....T...................HllLkn.........D.K........V.....LTLINTK.....IK** 157

**WP_009782430.1**  191 **..............................RLA..Q...--V.....SRIKI.....A...................-..-...........N.H........I.....LILINVH.....LE** 213

**PCJ61273.1**  94 **..............................LEK..E...RGF.....FWVRL.....K...................M..Kan.........D.K........S.....IFISTAH.....FT** 122

**XP_001022028.3**  151 **dldqsssiyselqw................INC..E...NIC.....LFTLL.....K...................D..Kkkp........N.S........F.....ILIGNTH.....FI** 194

**OMJ92191.1**  100 **..............................---..L...RVA.....Q--IL.....K...................L..Qkq.........N.Q........A.....FIFVNTH.....LD** 123

**XP_005836066.1**  93 **..............................MYV..D...GLL.....PAVPA.....D...................I..Ecpwevsrk...R.K........N.....TCIFLRL.....YH** 127

**XP_023241899.1**  827 **etssl.........................ENA..D...KIA.....IRTFL.....Qkcgyvl.............M..Mqfctvgnd...H.T........L.....TI---AN.....VF** 869

**RPD82043.1**  167 **gdeqarrgssf...................RTK..-...NIG.....SLVAL.....R...................Q..Lgsa........E.D........G.....VIVATTH.....LF** 206

**KXJ22915.1**  110 **dy............................TTF..K...KRS.....SFAVL.....S...................Y..Dls.........D.E........K.....FVVISWHgeykgLD** 145

**ORY43732.1**  132 **..............................TQK..D...NVA.....LLAVL.....Q...................L..Rpfksqttp...P.-........-.....-------.....IS** 157

**WP_053597247.1**  111 **..............................--LkaY...AKW.....IEINI.....E...................L..H...........QfK........K.....FNVIGVH.....VP** 138

**RJQ10437.1**  137 **..............................RDG..D...RGV.....LMVAV.....E...................T..Pl..........G.I........A.....-RIVNTH.....L-** 162

**WP_082554083.1**  380 **y.............................RGD..Q...KPM.....VWAVL.....R...................N..Rvt.........G.E........R.....WLCVSYH.....LE** 409

**OAQ31744.1**  250 **..............................TKK..E...NIG.....IVGIF.....D...................I..Khqer.......T.R........R.....VSLATSH.....II** 280

**XP_022302311.1**  326 **..............................--E..D...GRT.....LKVNF.....M...................H..R...........G.Kt.......N.....LTLICVY.....AP** 351

**CUF62170.1**  136 **..............................RFD..DgtsSQF.....AICVK.....L...................R..Vtai........D.T........D.....VIVVGHH.....AK** 168

**WP_010876562.1**  82 **..............................--I..E...DFY.....VSCRI.....E...................I..G...........G.N........S.....LSLFSIY.....NY** 106

**EWM30668.1**  260 **..............................GSR..-...-VG.....LLMDL.....E...................T..Lagatseqe...G.Q........R.....IILLSTH.....LS** 292

**position**  . 100 . 110 .

**ETO10795.1**  211 **GKPS..............K..H.D........I....RF..SQMKSLLQRL............DL..LFHeqnfp......KEKI...P..V.IICGDFNSTCDE...........VVVQMLl** 265

**PRP87210.1**  191 **GHPD..............A..T.D........K....RF..SEMKSLLDQM............QK..ELKkidsg......ETET...P..L.FIVGDFNCSIRS...........GIHTMLt** 245

**GT049204.1_EST**  189 **ASPD..............K..A.N........V....RL..SQVKKAVHSI............ER..QIAesehd......FGAF...P..I.ILCGDFNDGDDS...........NVADFMy** 243

**XP_027023568.1**  431 **WHPK..............G..A.N........V....RL..IQMAVALKHL............KK..VVMe..........EQAS...T..L.IFCGDFNSTPSS...........GLFQLLs** 481

**OAF70069.1**  729 **----..............Y..A.D........F....--..---VNFNKQV............FE..VISaveylkqfk..SNNV...P..I.VICGDFNSVPES...........AMYSFMt** 778

**XP_001320460.1**  307 **WEQT..............G..NdD........V....RT..SQLYLALEAA............KN..IVKqh.........SSNY...D..I.IIAGDFNSESQT...........TPHRWL.** 358

**RMZ57518.1**  653 **ASPL..............L..P.G........S....RL..HQLKAILGLL............--..-SEklgsrkaaa..AAQ-...-..L.VLAGDLGSTRQA...........SPCAFLs** 706

**XP_013759925.1**  203 **GHPA..............E..V.N........T....RL..SQVRSSLH--............KG..VLAgartfdaaat.LANL...R..L.VYCGDFNDEADS...........LVYKLMv** 260

**OLP93574.1**  1681 **AGPS..............-..A.D........R....RL..RQVHEALETV............EK..EAKrmska......PGEF...A..V.LVCGDFNSQGST...........AVRELLv** 1734

**XP_018711147.1**  146 **CGEH..............Q..S.K........S....RT..RQTAALMKSI............KE..FSEslgaeng....YSDC...Y..T.FLAGDFNSGPSS...........SAYQSLv** 202

**XP_002682001.1**  321 **GDPK..............Q..L.D........-....--..IQT-----EQ............AK..VLAnrsdmtkle..CACE...N..V.IIGGDFNASQES...........QAYQVV.** 370

**OJY24566.1**  128 **WEPE..............GtpP.D........A....HRgrVQLAELLD--............--..--T...........WPSG...P..R.IVCGDFNAEPDS...........DVL---.** 171

**RKP20909.1**  152 **FNSS..............R..G.Y........I....KL..IQMNMLLENI............NK..LFKkhel.......SSNC...P..I.FIMGDFNMTPFS...........GLYKFMv** 205

**XP_005778738.1**  364 **----..............-..-.-........V....RL..MQSAALVAEVeaeveaeaeaeaEA..EAEaeaeaeaea..GGEV...G..V.VVMGDLNSDASD...........GVLDLLl** 427

**XP_001749499.1**  741 **S---..............-..-.-........-....KR..SAEADRIKQM............RN..VVRlaeg.......MGTT...A..V.VLVGDFNDGDED...........PSVAQSl** 787

**OGN55659.1**  158 **WSK-..............-..E.K........TpsgpQW..NHVQFILQSI............PQ..TA-...........----...-..T.VVVGDFNMESDH...........PFM---.** 199

**WP_009782430.1**  214 **AFD-..............-..Q.P........T....RL..KQ----SQFV............LElfLSF...........SQKY...P..V.LLIGDFN----S...........EPPSEI.** 254

**PCJ61273.1**  123 **WQG-..............-..S.PdeketglsP....RI..AQTKRTIEFL............QK..LV-...........-KKD...D..VsFFMGDLNDPV--...........VPV---.** 171

**XP_001022028.3**  195 **YSPQ..............M..G.L........V....KL..GQAKLITSAI............KS..ILEaeg........DKNI...D..V.FLCGDFNFIPNS...........ALYSFFt** 247

**OMJ92191.1**  124 **HLS-..............-..E.S........I....RE..QQLRELMQAL............KP..YY-...........--DY...P..I.ICTGDYNFLPEV...........NNY---.** 164

**XP_005836066.1**  128 **MPCV..............F..W.D........Q....RV..MVIHSALAAR............HV..QSL...........SGAD...P..Y.VFAGDFNIQPQS...........AAYRLLt** 177

**XP_023241899.1**  870 **FPPS..............D..I.S........T....HA..LQMSSLVREV............VN..FAA...........DIAR...P..H.ILCGSFNMKEGG...........VAYQLLr** 919

**RPD82043.1**  207 **WHPA..............Y..A.Y........E....RA..RQAAILLREV............TK..FRSegtep......EARW...P..S.IIAGDFNFQPDD...........PAYSLLa** 261

**KXJ22915.1**  146 **CDK-..............-..K.E........Q....KV..RDLLSVLSQV............HS..-R-...........-TKL...P..I.ILAGDFN-----...........IAL---.** 181

**ORY43732.1**  158 **FSP-..............-..-.-........-....--..----------............--..LISypceqlskn..YGHL...S..T.IITGDFNMTEES...........VMYHYMr** 197

**WP_053597247.1**  139 **L---..............-..-.E........S....--..EKRIQFQKKF............QE..KFD...........L---...P..T.VLIGDFNA-ATN...........DDRAIEl** 177

**RJQ10437.1**  163 **---Tylr...........D..V.D........T....AV..LNLRQVRAVL............AE..PWL...........RDAA...V..IrLLCGDFNSEPDS...........DTIRYI.** 212

**WP_082554083.1**  410 **NESG..............A..D.L........A....RV..HQIAAIFAKV............AR..LR-...........-GQYgvaPdhV.VVGGDANSR---...........AWV---.** 455

**OAQ31744.1**  281 **FHPT..............Y..G.M........T....KI..AQLRMLLDSA............RE..MIVeq.........NSDI...P..I.VLCGDFNALPYS...........TVMRYLt** 332

**XP_022302311.1**  352 **N---..............-..-.N........-....-P..KERTFYFLRL............CD..LIRkeq........KKCQ...S..I.IICGDFNCDLNY...........DSDQSA.** 396

**CUF62170.1**  169 **AGRTeenettrichstelW..R.R........L....CA..LYAQD-----............DA..FLK...........GR--...-..L.LLCGDFNAGPHSyegkypaswypSIVEGLw** 235

**WP_010876562.1**  107 **I---..............-..-.G........P....AD..SDFSEFLDLL............YN..YIE...........EGED...L..I.IIGGDFN-----...........INKNFSp** 146

**EWM30668.1**  293 **FPHN..............R..F.D........E....N-..-QQHRQVSSL............TR..MLEayvkgcpalggQRDV...L..Q.ILCGDFN---VD...........LTNPVL.** 347

**position**  . 130 . 140 . 150 . 160 .

**ETO10795.1**  266 **nrkvdpnhsalle.................................SDDRN................FV.........HLYYF.R..EGYDE........V........FKKNR.H...IT..** 305

**PRP87210.1**  246 **egglaanfvdeesekph.............................NPGRE................YK.........HPFQL.K..NSYLE........Ak.......EIP--.-...-K..** 286

**GT049204.1_EST**  244 **gkleeqhqfvf...................................EDVEG................FM.........RRFCT.A..DADQK........Tl.......PSYFTqN...AA..** 283

**XP_027023568.1**  482 **qgsvleqhsdwasngpeeh...........................LP-ME................LH.........SPFQL.T..SAC--........G........EPQYT.N...YV..** 524

**OAF70069.1**  779 **tgkvskdqilqlrksydavnvddekkslld................FIPDE................MN.........QGIKF.T..DSYAN........Yv.......NNIGT.A...YNsk** 838

**XP_001320460.1**  359 **..............................................VQNGF................FD.........VYDSF.G..-----........L........SPRFT.I...YG..** 380

**RMZ57518.1**  707 **sgrvdahwteaglpkvg.............................LNRES................LE.........HAFPL.Q..EAYQA........Gq.......RLPQG.K...AQ..** 751

**XP_013759925.1**  261 **tgsvdagevqtfdgfddv............................VVGKA................AT.........HDASL.V..SSYAH........-........-CTGT.E...PT..** 303

**OLP93574.1**  1735 **ngivnpdfresgdptekgqegkq.......................ITSKI................RK.........HGLPT.F..QDAAD........Va.......FSGRA.P...AT..** 1785

**XP_018711147.1**  203 **skpfefspdtnwdssiqakieqahnridlrat..............SLYSV................GY.........HLVNL.Rn.SGIDN........Drn......EPPFS.F...WG..** 264

**XP_002682001.1**  371 **..............................................EQAGF................--.........-----.K..SAIKE........Ihgk.....EPTIT.F...NT..** 393

**OJY24566.1**  172 **..............................................----V................LA.........RSRGL.E..DAYAS........L........PDAYT.C...NS..** 194

**RKP20909.1**  206 **egfidlaqsgkenqlsgqnc..........................GRSQE................IKkywlnqtfeNPFSL.K..SS-MF........Mh.......ENSPD.NvsiFH..** 264

**XP_005778738.1**  428 **rgrvaashrdwragasprlpladadaakamarleelrarlrehgvsPPSPGvageptpdplppcgaaLC.........HSLDL.A..SAYG-........Ph.......CVP-T.H...CV..** 515

**XP_001749499.1**  788 **qcvrkledpva...................................AKAMV................AA.........ESAQL.L..LHSVY........Eg.......SPDVT.L...CK..** 826

**OGN55659.1**  200 **..............................................----Q................DF.........YSAGL.Q..DRFP-........-........---NT.F...SC..** 217

**WP_009782430.1**  255 **..............................................-NLEP................TI.........NLFLN.T..PGIQS........A........FPPKT.L...NH..** 280

**PCJ61273.1**  172 **..............................................----L................FF.........PGINY.K..SCFKE........Lgilsp...PTYPA.L...PT..** 199

**XP_001022028.3**  248 **qqsinfeslplhevsnqdmaytfe......................KNENS................ID.........QHFQ-.-..---ST........Tr.......KYQFF.N...KN..** 294

**OMJ92191.1**  165 **..............................................----K................-L.........MSKEF.I..SAYFQ........IhdkepmitFPTGL.Y...GP..** 194

**XP_005836066.1**  178 **sgrleashpdfppdra..............................GDSWA................PQ.........LEERM.E..SVYSS........Fhgs.....EPDFT.N...YAqi** 225

**XP_023241899.1**  920 **egylnnemieelqkrkdvalpgkdndtlvnl...............LWKAF................QH.........PSSNL.R..SCYATvlghelhlDg.......ENSIT.S...--..** 984

**RPD82043.1**  262 **gepllpg.......................................QKARL................EA.........SRVVH.A..TIDPS........Ia.......VEGAA.Q...AE..** 296

**KXJ22915.1**  182 **..............................................----E................KI.........KNIVIgS..DCWR-........-........-----.Y...YT..** 198

**ORY43732.1**  198 **tgetepflfhenys................................SGQNR................IT.........ANGNP.T..PYCYP........Yd.......ILEER.K...HI..** 239

**WP_053597247.1**  178 **lnksrlnen.....................................TNLLN................NI.........LNNNF.V..DSWRE........Vngkd....INEYT.W...FN..** 217

**RJQ10437.1**  213 **..............................................-REQS................GW.........HVQEA.TpkHPRG-........G........ERPVT.F...SP..** 239

**WP_082554083.1**  456 **..............................................----Rdw..............VA.........ENTDY.R..DAFDV........Aatvrd...KGIAS.I...NR..** 485

**OAQ31744.1**  333 **desidvsalpewr.................................LSGQQ................QA.........HATYK.F..TPPYE........Nk.......LTAFH.R...AF..** 373

**XP_022302311.1**  397 **..............................................ETLRH................VI.........RQCSL.I..DAWRH........Lqgn.....NQGHT.Y...V-..** 425

**CUF62170.1**  236 **gaegglvsaq....................................RSLVG................AE.........GEAQS.V..TTF--........-........---KL.R...KG..** 266

**WP_010876562.1**  147 **slrrlalla.....................................EEMTG................RL.........SELGF.R..DVLRE........E........EDPFT.F...MT..** 182

**EWM30668.1**  348 **..............................................APIHA................LG.........FQHVR.E..EGERG........Dg.......ADFVT.H...RT..** 375

**position**  170 . 180 . 190

**ETO10795.1**  306 **...F.-......-AANG......IlssIDFIFY..........TDN..N-.LL......LC......G......V..KKt.L.P.......DNiRPtrdnllllpn..A.RF.....P.....** 354

**PRP87210.1**  287 **...F.-......-TFKGknhrdsF...IDFIFH..........ST-..--.-S......AT......Q......V..AE..P.G.......DI.NHmrrnwlpn....E.EE.....P.....** 331

**GT049204.1_EST**  284 **...Y.-......-E---......-...LDFIFY..........TKS..N-.LL......PV......G......A..LH..G.L.......KG.HSdlqrmlkdpp..P.DAlg...P.....** 325

**XP_027023568.1**  525 **...G.-......-GFNG......C...LDYIFI..........ESQ..A-.LQ......VE......Q......V..IP..L.P.......SH.QEvttyqalps...V.SH.....P.....** 567

**OAF70069.1**  839 **wekF.-......-DYMK......T...IDYIFY..........EED..K-.LD......LV......G......I..MK..T.T.......AK.KHffnkkhips...R.CV.....P.....** 884

**XP_001320460.1**  381 **...A.-......-SFHK......T...IDFIYS..........TVN..K-.IR......PI......S......I..LQ..S.Y.......NE.EEilqryvafph..D.YF.....P.....** 424

**RMZ57518.1**  752 **...V.-......-PYRD......T...-DFIWC..........SRG..G-.-R......VV......A......V..LD..PmP.......AG.LRhgaphaslpn..S.TV.....P.....** 794

**XP_013759925.1**  304 **...V.-......-TFKApnmrtsT...IDFVFA..........ALP..H-.LR......PL......M......V..FD..R.L.......ST.PEaiadvirtslpsA.EH.....P.....** 355

**OLP93574.1**  1786 **...I.-......-LAQN......I...DDKMVN..........PDG..T-.LT......ES......M......G..KA..L.D.......AA.FD............A.CR.....S.....** 1819

**XP_018711147.1**  265 **...P.-......-DNRG......L...LDYIFVicrwsgddkkGVD..T-.LE......EL......SeagdvkL..LG..L.L.......RM.PRaeemygsipq..VgMY.....P.....** 325

**XP_002682001.1**  394 **...D.-......-AIHR......S...IDYLWY..........NCS..T-.IM......PS......T......C..TI..F.N.......QK.QIeikkeealpn..H.QY.....G.....** 437

**OJY24566.1**  195 **...N.-......-DKKK......R...IDFLLH..........SSD..F-.VA......TP......S......P..TP..P.V.......DD.DTplps........E.LE.....P.....** 232

**RKP20909.1**  265 **...L.-......-GSES......I...VDYIFY..........GSI..K-.EV......GD......K......I..LP..V.Q.......GK.EFlekrnmksflpnE.DF.....P.....** 310

**XP_005778738.1**  516 **...A.-......-GYAN......A...LDWICF..........SRE..T-.LR......VV......G......VapLP..P.I.......DE.LRrhvaips.....A.EF.....P.....** 558

**XP_001749499.1**  827 **...P.-......-GLMA......R...IDHVLV..........TSN..C-.LV......EA......R......H..AP..Q.A.......TE.SYgtlskhrlps..R.RN.....G.....** 870

**OGN55659.1**  218 **...Y.A......NKNFQ......K...IDYILS..........TND..L-.-Q......NT......P......F..ES..T.S.......LS.EA............Q.LLpneneP.....** 257

**WP_009782430.1**  281 **...P.EngtypsDQPHR......T...IDYIFY..........NQN..KIeAI......EW......Q......V..VT..D.I.......LA.--............-.--.....-.....** 318

**PCJ61273.1**  200 **...T.Nd.....ICENQ......A...IDWMFC..........NGK..A-.-K......AL......S......A..FV..P.Q.......FY.FD............G.IS.....P.....** 235

**XP_001022028.3**  295 **...E.-......-TQKE......Y...IPY-FQ..........TLM..S-.LE......VK......F......D..KK..N.Q.......EF.NVvfnkneniks..K.NI.....I.....** 337

**OMJ92191.1**  195 **...Y.Ad.....IESYG......C...FDYIWI..........R-G..A-.-K......AI......S......A..EV..Y.R.......DC.GNq...........Q.IW.....A.....** 230

**XP_005836066.1**  226 **fedP.-......-PFIE......T...IDYIF-..........CRP..G-.MK......VV......G......C..LP..L.P.......RR.QEvsgplpn.....D.QE.....P.....** 268

**XP_023241899.1**  985 **...-.-......-----......-...PDLLWY..........SSD..S-.LE......TI......G......I..LDfvP.V.......DK.NN............I.DP.....V.....** 1014

**RPD82043.1**  297 **...E.-......-EEEG......A...ESGEND..........PDR..I-.IV......NA......R......A..AQ..P.S.......DG.LM............T.DD.....E.....** 330

**KXJ22915.1**  199 **...Y.El.....GNRST......R...IDYFVV..........TRD..F-.-V......LS......D......I..EA..L.T.......DY.RY............V.TN.....E.....** 234

**ORY43732.1**  240 **...R.-......-SSGD......A...SKCIPL..........IRP..Q-.AA......AD......Q......R..VL..D.F.......CG.GS............D.GL.....M.....** 273

**WP_053597247.1**  218 **...SmD......KNDGR......R...LDYAFV..........TNN..IE.ILavnhlpEL......N......M..IL..D.E.......NG.--............-.--.....F.....** 256

**RJQ10437.1**  240 **...R.Nanvre.GARSV......V...LDYVFS..........LAPaaGRqPR......MS......S......A..VV..L.D.......QP.TG............S.VF.....P.....** 284

**WP_082554083.1**  486 **...W.Kv.....PAAGE......R...EDAVFV..........HKT..A-.-D......VE......-......-..--..L.A.......DQ.RD............G.HK.....S.....** 517

**OAQ31744.1**  374 **...P.-......-DNGH......G...FSPTSK..........AFP..V-.AS......PT......R......T..KS..D.P.......TA.AA............A.TT.....T.....** 407

**XP_022302311.1**  426 **...-.R......GSSHR......R...IDYVFL..........TEN..LI.FP......SF......N......D..MS..D.T.......GT.KT............V.LF.....P.....** 461

**CUF62170.1**  267 **...A.-......-WIEQ......C...IDYVFH..........NPS..G-.LA......AC......G......G..LQ..L.PcqltaakGV.EEvnaaptph....A.LL.....Pqeglp** 320

**WP_010876562.1**  183 **...P.N......G-GKY......Q...IDYLFV..........PEG..VK.IL......DVwhppagE......I..IE..T.R.......PR.--............-.--.....L.....** 219

**EWM30668.1**  376 **...H.-......-RGDD......V...AVDHIF..........YRS..F-.NA......AR......H......R..RR..G.A.......TG.NT............S.EE.....D.....** 409

**position**  . 200 . 210 . 220 . 230

**ETO10795.1**  355 **......SDHLPIAALF** 364

**PRP87210.1**  332 **......SDHLPVASIF** 341

**GT049204.1_EST**  326 **......SDHMPVGAFY** 335

**XP_027023568.1**  568 **......SDHIALVCDL** 577

**OAF70069.1**  885 **......SDHLSLKSVF** 894

**XP_001320460.1**  425 **......SDHLPIVACF** 434

**RMZ57518.1**  795 **......SDHTPRGAIL** 804

**XP_013759925.1**  356 **......SDHLPVCAVI** 365

**OLP93574.1**  1820 **......GNEMTAGETE** 1829

**XP_018711147.1**  326 **......SDHLCMMADL** 335

**XP_002682001.1**  438 **......SDHIPIFAKF** 447

**OJY24566.1**  233 **......SDHLPIEARF** 242

**RKP20909.1**  311 **......SDHVPL---V** 317

**XP_005778738.1**  559 **......SDHVSLVADL** 568

**XP_001749499.1**  871 **......SDHLPVGMDF** 880

**OGN55659.1**  258 **......SDHLPLGSVI** 267

**WP_009782430.1**  319 **......SDHLPVVMKF** 328

**PCJ61273.1**  236 **......SDHWPIHAFY** 245

**XP_001022028.3**  338 **......TSDICLKSAY** 347

**OMJ92191.1**  231 **......SDHYPVYGEI** 240

**XP_005836066.1**  269 **......SDHIMIGADF** 278

**XP_023241899.1**  1015 **......ALKADIAYIC** 1024

**RPD82043.1**  331 **......LEQLYRQLGT** 340

**KXJ22915.1**  235 **......SREARPNSSP** 244

**ORY43732.1**  274 **......RHNFKFEDAI** 283

**WP_053597247.1**  257 **......TDHSGIEIKI** 266

**RJQ10437.1**  285 **......SDHFGVYAEV** 294

**WP_082554083.1**  518 **......SDHNPQVVDV** 527

**OAQ31744.1**  408 **......RTNATETATT** 417

**XP_022302311.1**  462 **......LSHISLHDSE** 471

**CUF62170.1**  321 **kvglwgSDHLSIAFDF** 336

**WP_010876562.1**  220 **......SDHIPLMVTL** 229

**EWM30668.1**  410 **......PRSRGPEGAT** 419

**position**  . 240
