# Supplementary figures and images for: Obtaining extremely large and accurate protein multiple sequence alignments from curated hierarchical alignments
Source: Database (Oxford). 2020 Jun 8;2020:baaa042. doi: 10.1093/database/baaa042 (PMC7297217; doi:10.1093/database/baaa042)

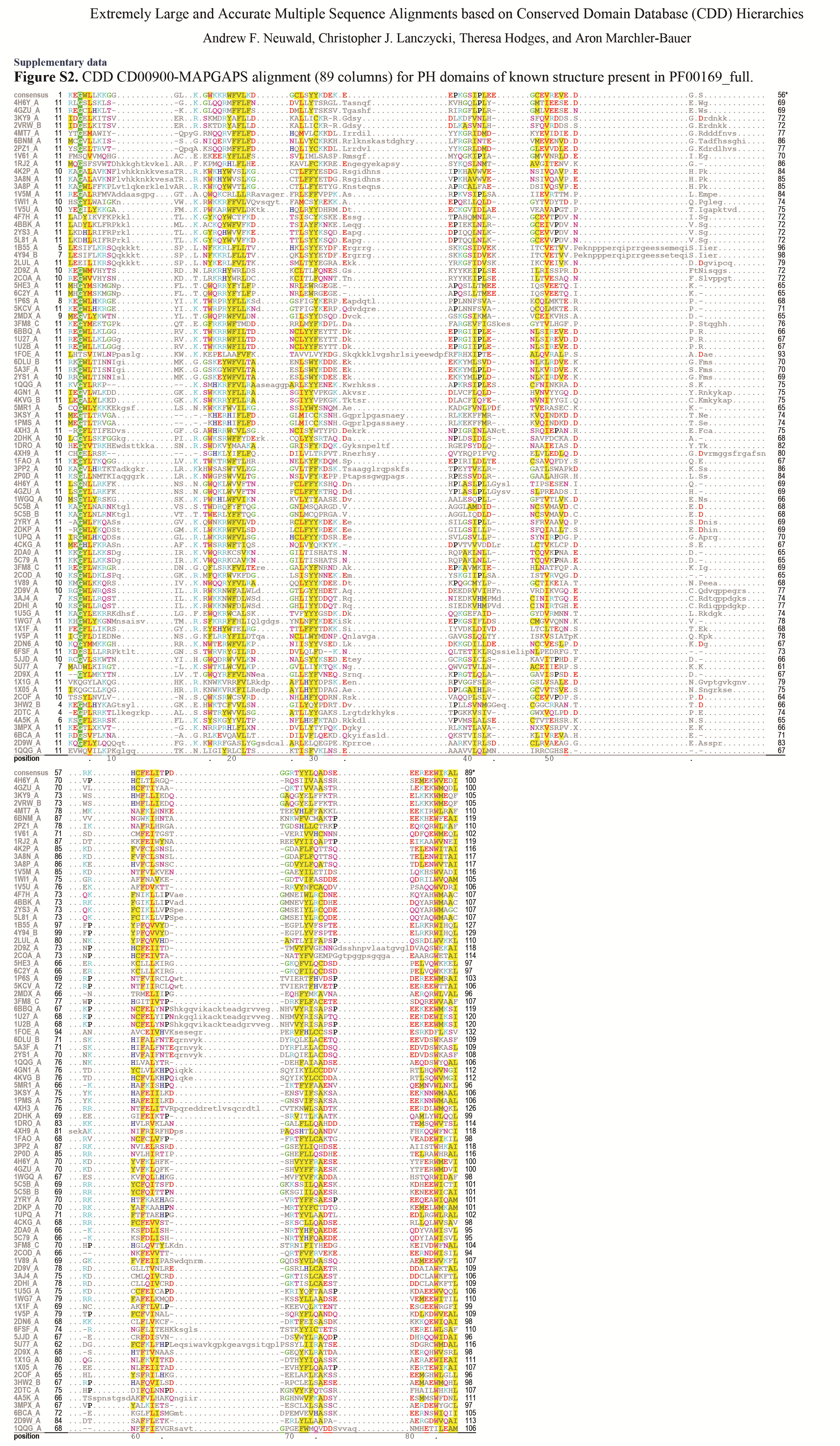

Supplement: neuwald_FigS2_baaa042 [file neuwald_figs2_baaa042.png]

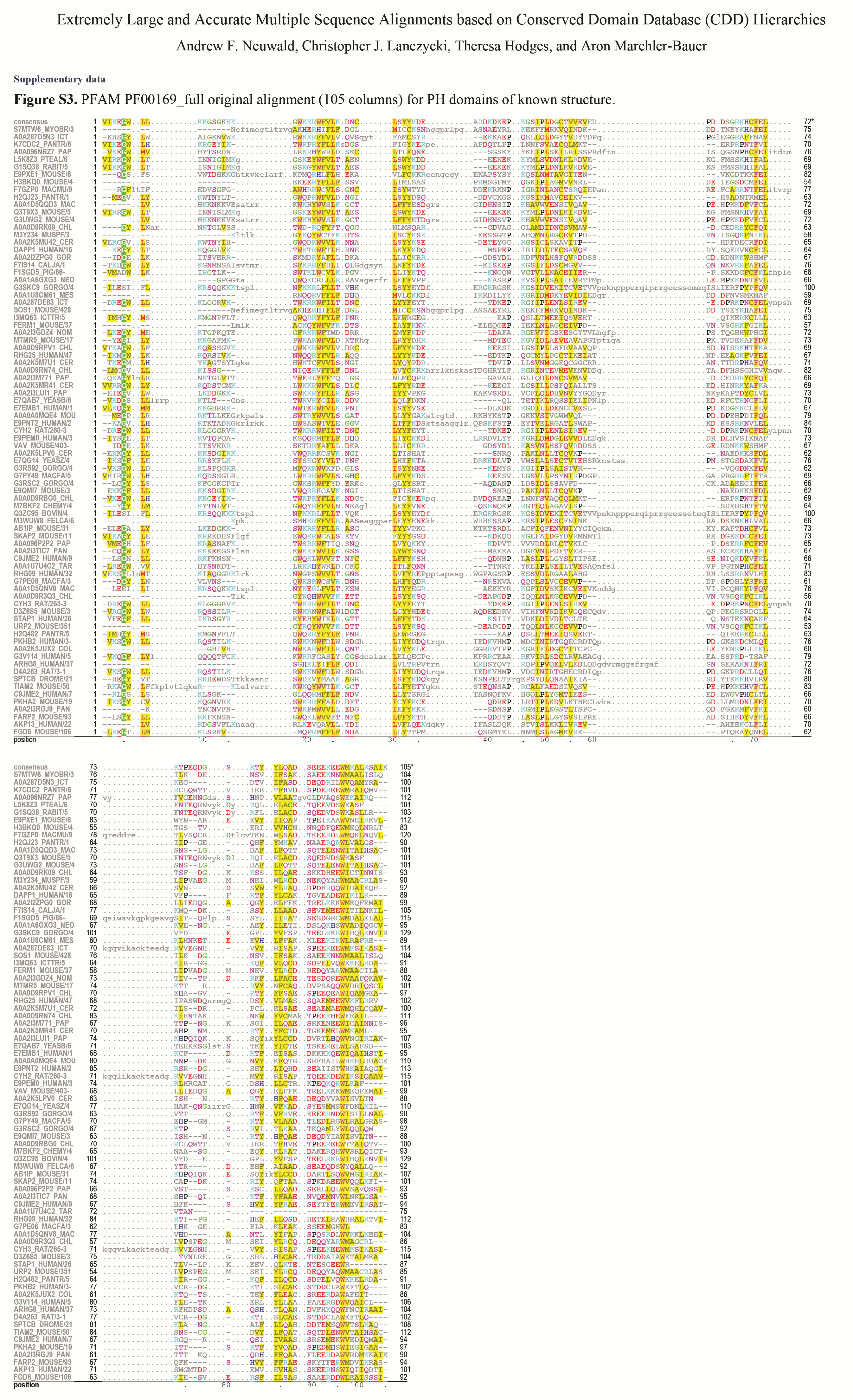

Supplement: neuwald_FigS3_baaa042 [file neuwald_figs3_baaa042.png]

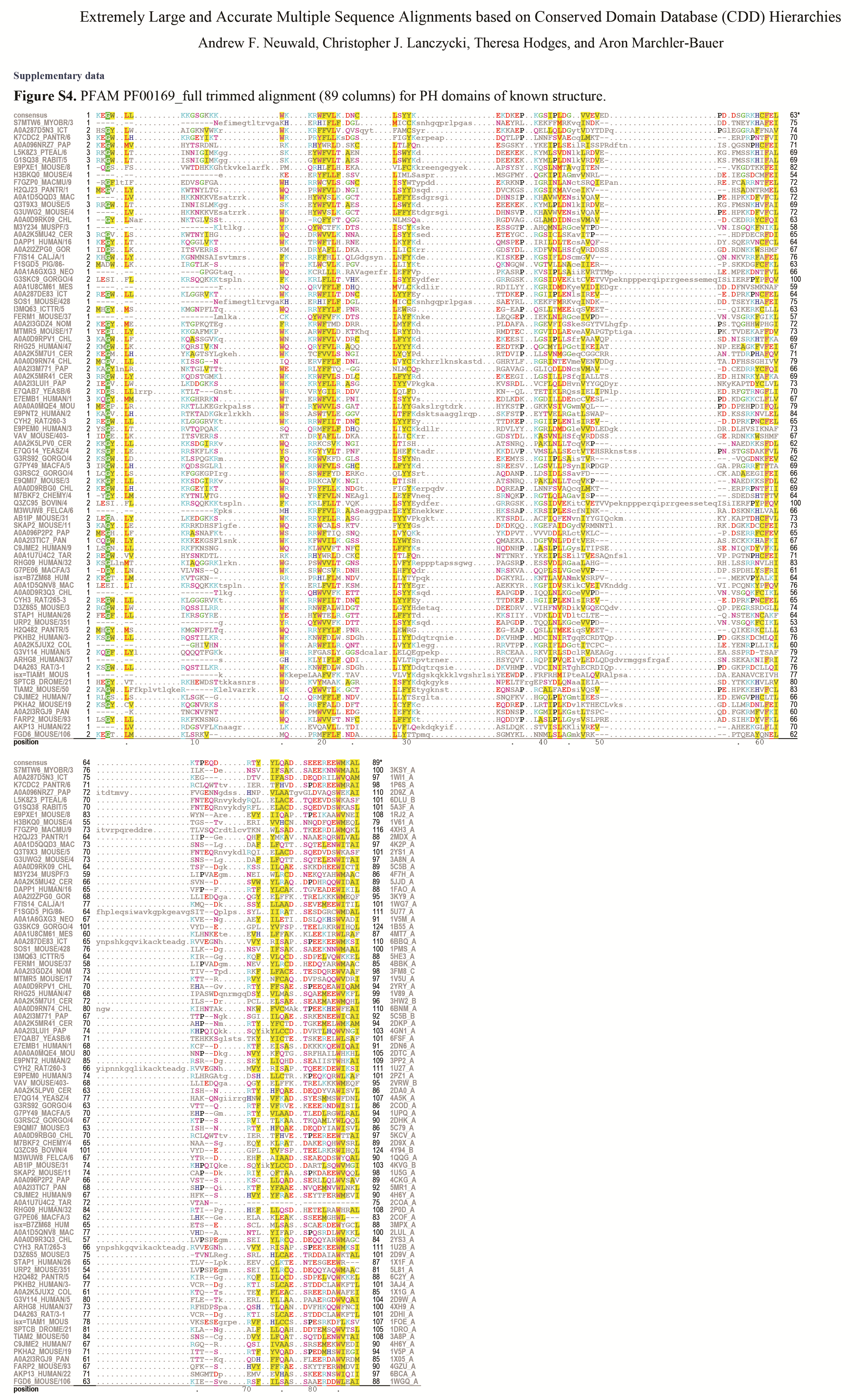

Supplement: neuwald_FigS4_baaa042 [file neuwald_figs4_baaa042.png]
