# Supplementary material for: Obtaining extremely large and accurate protein multiple sequence alignments from curated hierarchical alignments
Source: Database (Oxford). 2020 Jun 8;2020:baaa042. doi: 10.1093/database/baaa042 (PMC7297217; doi:10.1093/database/baaa042)
Supplement: neuwald_TableS2_baaa042 [file neuwald_tables2_baaa042.docx]

# Obtaining Extremely Large and Accurate Protein Multiple Sequence Alignments from Curated Hierarchical Alignments

## Andrew F. Neuwald, Christopher J. Lanczycki, Theresa Hodges and Aron Marchler-Bauer

### **Supplementary Tables**

**Table S2**. Semantics of taxonomy annotated variables added to fasta-formatted sequences definition lines by the AddPhylum program.

|  | variable | type | semantics | example |
| --- | --- | --- | --- | --- |
|  | *seqid* | string | sequence identifier | RPF34781.1 |
|  | *offset* | integer | start site offset | 0 |
|  | *taxid* | integer | species identifier | 2485151 |
|  | *phylum* | string | phylum | Actinobacteria |
|  | *kingdom* | character | major group^a^ | B |
|  | *description* | string with spaces | protein description | ATP-binding cassette… |

^a^The letter designations for kingdoms are: metazoa, M; protozoa, E; plants, V; fungi, F; bacteria, B; archaea, A.
